# GLP and G9a histone methyltransferases as potential therapeutic targets for lymphoid neoplasms

**DOI:** 10.1186/s12935-024-03441-y

**Published:** 2024-07-12

**Authors:** Amandda Évelin Silva-Carvalho, Luma Dayane Carvalho Filiú-Braga, Gabriela Muller Reche Bogéa, Alan Jhones Barbosa de Assis, Fábio Pittella-Silva, Felipe Saldanha-Araujo

**Affiliations:** 1 Hematology and Stem Cells Laboratory, Faculty of Health Sciences, University of Brasília, Brasilia, Brazil; 2 Laboratory of Molecular Pathology of Cancer, Faculty of Health Sciences and Medicine, University of Brasilia, Brasília, Brazil

**Keywords:** GLP, G9a, Lymphoid neoplasms, Acute lymphoblastic leukemia, Multiple myeloma, Chronic lymphoblastic leukemia

## Abstract

Histone methyltransferases (HMTs) are enzymes that regulate histone methylation and play an important role in controlling transcription by altering the chromatin structure. Aberrant activation of HMTs has been widely reported in certain types of neoplastic cells. Among them, G9a/EHMT2 and GLP/EHMT1 are crucial for H3K9 methylation, and their dysregulation has been associated with tumor initiation and progression in different types of cancer. More recently, it has been shown that G9a and GLP appear to play a critical role in several lymphoid hematologic malignancies. Importantly, the key roles played by both enzymes in various diseases made them attractive targets for drug development. In fact, in recent years, several groups have tried to develop small molecule inhibitors targeting their epigenetic activities as potential anticancer therapeutic tools. In this review, we discuss the physiological role of GLP and G9a, their oncogenic functions in hematologic malignancies of the lymphoid lineage, and the therapeutic potential of epigenetic drugs targeting G9a/GLP for cancer treatment.

## Introduction

Hematological malignancies are among the most common types of cancer worldwide. Lymphoid neoplasms represent a heterogeneous group of diseases that can derive from B, T, or NK cells. In general, the incidence of lymphoid malignancies has increased in recent years [1, 2]. According to the Surveillance, Epidemiology, and End Results (SEER) registry, the 5-year age-adjusted incidence rates (2017–2021) of multiple myeloma (MM), chronic lymphocytic leukemia (CLL), and acute lymphocytic leukemia (ALL) were 7.2, 4.6, and 1.8 per 100,000 people, respectively. Despite the important advances achieved recently in the treatment of some lymphoid neoplasms, such as the use of new drugs such as tyrosine kinase inhibitors [3, 4] and the development of CAR-T cells [5], several aspects highlight the need for other therapeutic approaches for these malignancies, including the toxicity and cost associated with available treatments, the high mortality of some subtypes of lymphoid neoplasms, in addition to the increasing knowledge about the genomic and molecular alterations of these diseases [2, 6]. Interestingly, lymphoid neoplasms present widespread epigenomic alterations, which makes these modifications important molecular targets for the development of personalized therapeutic approaches [7].

In fact, accumulating evidence has demonstrated the impact of epigenetics on cancer development and progression. Alterations in DNA methylation patterns and modification in histone residues can affect the chromatin transcriptional profile, repressing or activating genes related to cell proliferation, DNA repair, differentiation, and apoptosis [8–11]. Chromatin is a macromolecular complex in which our DNA is packaged, and its structure has a large impact on gene regulation process. The nucleosome is the basic unit of chromatin, composed of approximately 147 base pairs of DNA and an octamer formed by two pairs of histones H2A, H2B, H3, and H4 associated with the binding histone H1 [11, 12]. Histone tails are subject to extensive covalent post-translational modifications that influence chromatin status. The main histone modifications are acetylation, phosphorylation, methylation, sumoylation, and ubiquitination [11, 13]. Additionally, histones can also be modified by citrullination, ADP-ribosylation, deamination, formylation, propionylation, and proline isomerization, among other modifications [11, 12, 14].

Methylation is one of the first described post-translational modifications of histones [15], that can either result in the activation or suppression of gene expression, depending on the modified amino acid residue [16]. Histone methyltransferases and histone demethylases are functionally antagonistic enzymes that regulate histone methylation and play an important role in controlling transcription by altering the structure of chromatin. Dysregulation in the activity of these enzymes can affect the methylation pattern of histones leading to vicious abnormal gene transcription. It has been widely reported that aberrant activation of histone-lysine methyltransferases (HMTs) in certain types of neoplastic cells can modify the expression of specific genes through increased methylation of key lysine residues [15, 17, 18].

**Fig. 1:**
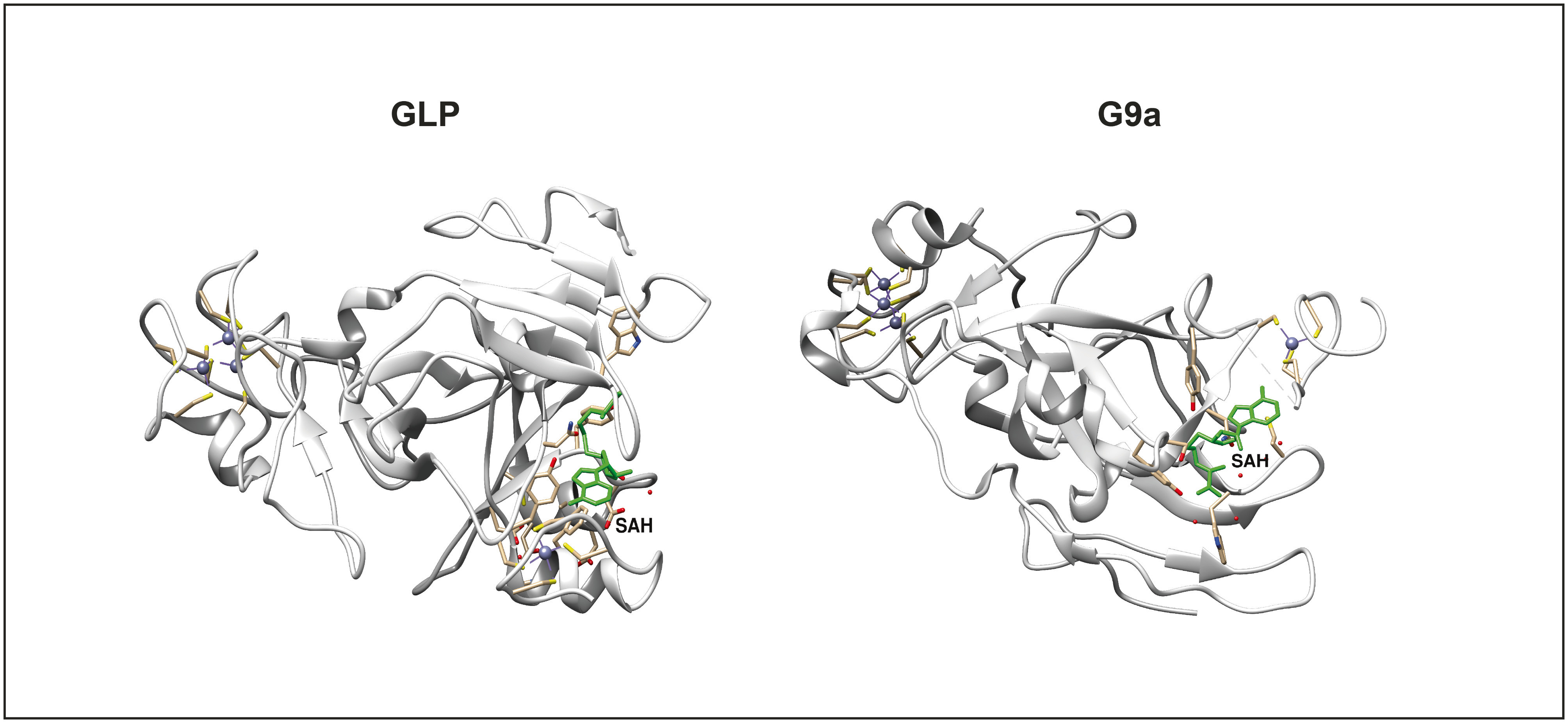
Protein structures of G9a and GLP. Crystal structure of GLP (PDB ID: 3FPD) and G9a (PDB: 208 J) with SAH bounded in green

Among the HMTs, G9a, also known as Euchromatic Histone Methyltransferase 2 (EHMT2), and its closely related partner, GLP (G9a-like protein or EHMT1), are crucial components of the histone methylation machinery. These enzymes mainly catalyze the dimethylation of lysine 9 on histone H3 (H3K9me2), a modification generally linked to transcriptional repression and the formation of heterochromatin. G9a and GLP together create a functional heteromeric complex crucial for their enzymatic activity in vivo [19, 20] (Fig. 1). Loss of either G9a or GLP leads to embryonic lethality in the mouse, demonstrating they play critical roles in development [21]. Dysregulation of G9a has been reported to be associated with self-renewal and tumor initiation in different types of cancer [18], and its overexpression has been linked with poor prognosis [22, 23]. In addition, G9a-mediated H3K9 di‐methylation silences the tumor suppressor genes, potentially increasing cancer cell proliferation [24, 25]. GLP functions in cancer cells are not well described and elucidated. However, GLP is overexpressed in lung cancer and modulates the expression of the CDKN1A gene, promoting cancer cell proliferation [26]. In human esophageal squamous cell cancer, GLP expression is elevated and may play a significant role in cancer progression [27]. Moreover, GLP knockdown in bowel cancer induces E-cadherin expression, resulting in decreased peritoneal metastasis in vitro and in vivo [28]. The key roles played by both enzymes in several diseases made them attractive targets for drug development. In fact, in recent years, several groups have tried to develop small molecule inhibitors targeting their epigenetic activities as potential antineoplastic therapeutic tools [29, 30]. In this review, we discuss the physiological role of GLP and G9a, their oncogenic functions on hematological malignancies from lymphoid lineage, the therapeutic potential of epigenetic drugs in cancer, and the scientific challenges that need to be overcome for epigenetic drugs to become a therapeutic option for cancer.

## Histone methylation and the physiological roles of GLP and G9a

Modifications on DNA and histones are dynamically established and removed by enzymes that modify chromatin in a highly regulated manner [12]. Histone methyltransferases play important roles in regulating transcription and responding to DNA damage [31, 32]. Histone methylation occurs at the amino acid residues of lysine or arginine with the addition of a methyl group (− CH3) [31]. The methylation of lysine in the ε-amino group is catalyzed by (HMTs, which can add a mono- (me), di- (me2), or tri (me3) the methyl groups, donated from S-adenosyl-L-methionine (SAM) to its target residue [31, 33]. On the other hand, histone demethylases (KDMs) mediate the removal of these groups [31]. A proper balance between HMTs and KDMs in regulating the methylation state of lysine residues is crucial for maintaining cell fate and genomic stability [34]. HMTs are divided into histone lysine methyltransferases (KMTs) and protein arginine methyltransferases (PRMTs) [35]. Currently, there are over 50 identified KMTs [36], but the number of KMTs in the human genome is predicted to be much larger [33].

Among the various sites of histone methylation, the most well-studied are those that occur at lysine residues, such as H3K4, H3K9, H3K27, H3K36, H3K79, and H4K20. Mutations in H3K4, H3K36, and H3K79 are generally considered active transcription markers, while markers in H3K9, H3K27, and H4K20 are associated with silenced chromatin [12, 37].

The histone methyltransferase G9a (coded by the *EHMT2* gene) forms a dimer with GLP (coded by the *EHMT1* gene) [20, 38]. The heteromeric complex formed is ubiquitously expressed and both enzymes are crucial for methylation at H3K9 in euchromatin regions [19, 39]. G9a and GLP belong to the SET domain KMTs family that add mono-, di- and trimethyl groups in histone H3, mainly in the K9 residue, in addition to the K27 residue and the histone H1 [19]. G9a and GLP share 45% sequence identity and about 70% similarity. They can form homo- or heterodimers through their SET domains. These proteins usually act as heterodimers in a multitude of human cells. However, G9a is the main methyltransferase that catalyzes mono- and dimethylation events at residues of H3K9 (basic substrate for both G9a and GLP) [17].

**Fig. 2:**
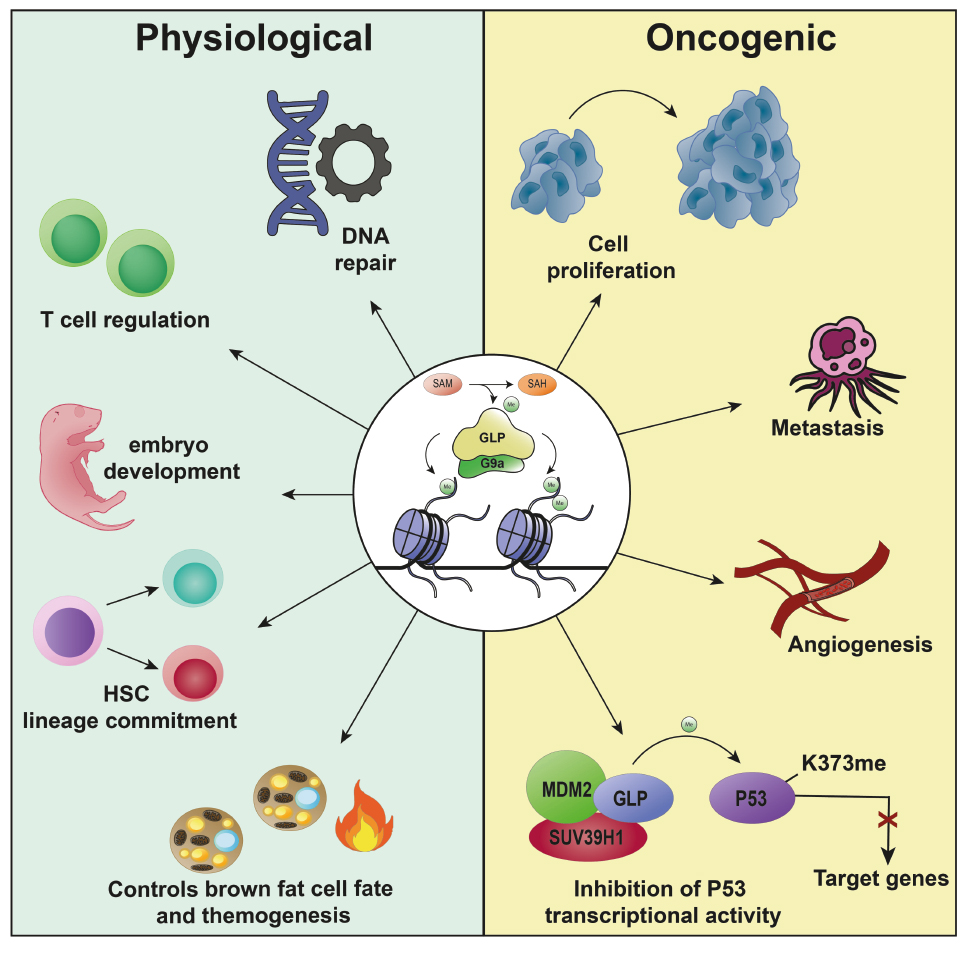
Schematic overview of G9a/GLP physiological functions and oncologic roles. G9a and GLP are involved in the regulation of several physiological processes (blue panel), including DNA repair, embryonic development, regulation of T cells, and impairment of the hematopoietic stem cell lineage. GLP also controls brown fat cell fate and adaptive thermogenesis. In the oncological context (yellow panel), they are related to the cell proliferation process, metastasis, and regulation of angiogenic factors. Additionally, GLP is involved in the repression of P53 activity through the MDM2/SUV3941/GLP complex, which induces the methylation of P53 lysine residue 373

Both G9a and GLP have been implicated in various biological processes beyond their canonical roles in transcriptional silencing, as illustrated in Fig. 2. These processes include a crucial role in embryo development, cell differentiation and growth, autophagy, adipogenesis, and other biological processes [19, 39, 40] (Fig. 2). It was shown that the G9a knockout impacted embryonic development causing growth interruption in the first stages, and embryos’ impracticality after a few days [21]. It was also suggested that methyltransferase G9a regulates cell proliferation and differentiation in the dental mesenchyme, being necessary for proper dental development [41]. In addition, G9a can also methylate non-specific histone proteins involved in DNA repair mechanisms, such as polo-like kinase 1 (Plk1) and p53 [17, 18, 42] and several other proteins, including G9a itself, CSB, histone deacetylase (HDAC) 1, KLF12, SIRT1, Reptin, MyoD, CDKN1A, among others [43].

DNA damage poses a threat to cell viability, compromising the integrity of the genome and the epigenome [44]. G9a and GLP are critical components of the DNA repair pathway, and they are rapidly localized to DNA breaks. The catalytic activity of G9a is required for the early recruitment of DNA repair factors, including 53BP and BRCA1 for DNA breaks. Inhibition of G9a catalytic activity interrupts DNA repair pathways and increases sensitivity to ionizing radiation [45]. Furthermore, G9a interacts with the replication protein A (RPA) to facilitate DNA repair via homologous recombination (HR) [46]. In colon cancer, downregulation of G9a has been shown to increase the rate of chromosomal aberration, and induce DNA double-strand breaks, which could trigger a response to DNA damage, cellular senescence, and tumor growth arrest [47].

Increased G9a activity also led to gene silencing under hypoxic conditions. In addition, G9a represses genes, playing a key role in cellular processes, especially in solid tumors where hypoxia is a common condition [17]. In embryonic stem cells, gene repression of pluripotency factors via G9a-dependent H3K9me2 is extremely necessary for the development of cell lines. In embryonic stem cells deficient in G9a, there is sustained silencing of genes associated with pluripotency, which results in the inversion of most cells from the differentiated to a pluripotent state [43].

Interestingly, G9a appears to act in the regulation of the immune system. In macrophages, G9a-dependent H3K9me2 was associated with gene repression during endotoxin tolerance [43, 48]. This highlights a role for G9a in gene silencing during cellular responses to inflammatory signals. In contrast, T lymphocytes from patients with type I diabetes exhibited a distinct H3K9me2 profile, with genetic regions that increased (*CXCL3*,* CTLA-4*,* SLC17A4*) and reduced (*RARA*,* CAMK4*,* TNF*) H3K9me2 levels [49], suggesting a G9a-mediated H3K9me2 dependent regulation in T cells, which may be associated with T lymphocyte function, as in the development of inflammatory diseases such as diabetes [43].

In Th1 cells that modules IFN-γ, cells targeting intracellular pathogens, the absence of G9a in T cells does not affect the development of Th1 cell responses in vitro or in vivo. In contrast, G9a-deficient Th2 cells express comparable levels of major Th2 cell transcription factors, including GATA3 and STAT6, but fail to produce type II cytokines [43, 50], suggesting that G9a is a central component of the machinery of transcription for type II cytokines. In B cells, G9a does not appear to play a direct role in normal B cell development and has little effect on most B cell functions [43].

On the other hand, little is known about the biological functions of GLP. GLP seems to be an essential enzymatic switch that controls brown fat cell fate, adaptive thermogenesis, and glucose homeostasis in vivo. Loss of GLP in brown adipocytes causes a severe loss of brown fat characteristics and induces muscle differentiation in vivo [51]. Constitutional deletions of distal 9q34 encompassing the GLP gene, or loss-of-function point mutations in GLP, are associated with 9q34.3 microdeletion syndrome, also known as Kleefstra syndrome, characterized by developmental delay, intellectual disability, and other variable clinical features [52–54]. In addition, in cancer, overexpression of GLP has been observed in colorectal cancer and suppression retards cell growth by inducing cell apoptosis in colorectal cancer cell lines [55]. In alveolar rhabdomyosarcoma (ARMS), GLP is expressed in both primary and recurrent tumors. GLP suppression impairs motility, induces differentiation into ARMS cell lines, and reduced tumor progression in a mouse xenograft model [56]. In oral squamous cell cancer, *in silico* analysis identified GLP among a group of 9 highly expressed genes that may influence the aggressiveness of the disease [57].

G9a and GLP also seem to play a crucial role in hematopoietic stem cell lineage commitment [58]. Chromatin Immunoprecipitation followed by sequencing (ChIP-seq) analysis and immunofluorescence (IF) staining revealed that H3K9me2 patterns promoted by G9a/GLP in human hematopoietic stem and progenitor cells (HSPCs) are progressive and associated with high-order chromatin changes during lineage specification. Accordingly, treatment with G9a/GLP inhibitor UNC0638 has been shown to help HSPCs retain their stem cell-like characteristics during in vitro expansion. This inhibition delays lineage commitment and maintains a more primitive state of HSPCs [58]. In addition, UNC0638 reactivated genes silenced by G9a in mouse embryonic stem cells, without promoting differentiation [59]. This indicates that G9a/GLP inhibition can maintain stem cell pluripotency while allowing the reactivation of key genes necessary for stem cell maintenance.

## Oncogenic functions of GLP and G9a in lymphoid neoplasms

Dysregulation in GLP and G9a is associated with oncogenesis and lymphoid neoplasms. As previously discussed, these HMTs act on biological processes that are altered in these pathologies, including DNA repair, cell growth, and cell death. In fact, over the last 20 years, numerous studies have correlated the epigenetic methylation pattern with cancer development [60]. In addition to DNA methylation, alterations in histones are also involved in carcinogenesis [10, 11, 61]. H3K9 methylation by G9a/GLP is usually associated with chromatin transcriptional inactivation and repression of tumor suppressor gene expression, leading to cancer initiation and progression [23, 39, 62].

Aberrant expression of G9a/GLP in cancer cells is commonly associated with a higher metastatic capacity and poor prognosis. G9a overexpression was observed in different types of tumors such as esophageal squamous cell carcinoma [63], melanoma [64], lung cancer [65, 66], and gastric cancer [67]. Likewise, GLP is upregulated in esophageal squamous cell cancer [27], ovarian carcinoma [68], and gastric cancer [28].

Alterations in epigenetic modifiers also suggested an important link between epigenetic modifications and leukemogenesis [69, 70]. Although leukemogenesis is usually associated with chromosomal abnormalities and mutations affecting genes that regulate self-renewal, proliferation, and differentiation processes [71], mutations in epigenetic regulators also seem to contribute to hematopoietic transformation in both myeloid and lymphoid malignancies [69, 72–75].

**Fig. 3:**
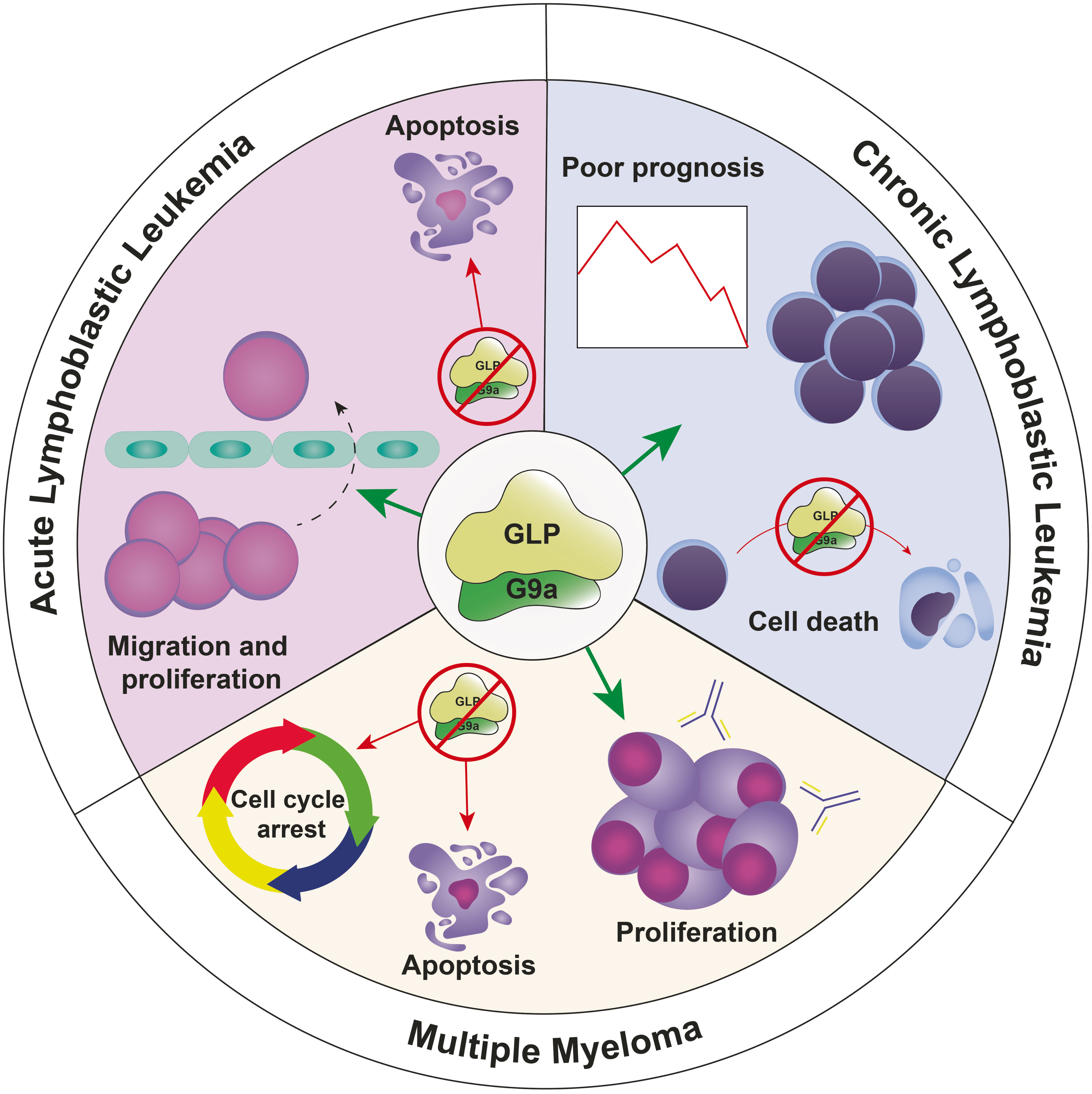
Schematic overview of G9a/GLP influences in ALL, CLL and MM. These HMTs control migration, proliferation, and apoptosis in ALL. In CLL, G9a is associated with poor prognosis, and its inhibition induces cell death. G9a contributes to MM cell survival and proliferation. G9a/GLP inhibition induces cell apoptosis and G1-phase arrest

G9a and GLP seem to play a critical role in several hematological malignancies such as multiple myeloma (MM) [76] and both myelocytic [77–79] and lymphocytic leukemias [80, 81] (Fig. 3).

## Acute lymphoblastic leukemia

ALL is a malignant hematopoietic disease originating from precursor cells of the lymphoid lineage (lymphoblasts) with predominant bone marrow and peripheral blood involvement and, in a few cases, even tissue infiltration [82, 83]. According to the World Health Organization classification (WHO), ALL comprises a group of neoplastic diseases that involves lymphoid lineage precursor cells such as B and T cells (B-ALL / T-ALL), with B-ALL encompassing approximately 85% of cases [84].

ALL accounts for less than 1% of all cancers in the United States, but it has a higher incidence in children under 5 years old with significant morbidity and mortality, being the most common pediatric cancer [82, 85, 86]. This neoplasm primarily presents an acute clinical development, and, in exceptional cases, it could be insidious. The most common symptoms are directly related to the hematological repercussion of bone marrow failure and include fever, fatigue, lethargy, bleeding disorders, bone, and joint pain [82].

Although ALL appears to be homogeneous when using morphological and immunophenotypic criteria, it encompasses a large level of heterogeneity when based on genetic factors involved [82, 87, 88]. The detection of genetic alterations is particularly relevant in ALL, as it allows classification into different subgroups of the disease [89, 90]. ALL is usually associated with chromosomal mutations, aneuploidy, and gene rearrangements resulting in translocation processes that express chimeric fusion proteins (e.g., BCR-ABL1, TCF3/PBX1, and ETV6-RUNX1), deletions, or gains of DNA [86, 91–94].

In addition to genetic events, changes in the epigenetic pattern of tumor suppressor genes or genes involved in signaling pathways for cell cycle, apoptosis, and cell damage response are essential for the development of ALL [91, 95–97]. DNA methylation, histone modification, and microRNA alteration are the main processes involved. Among them, DNA hypermethylation in promoter regions is the most described. Moreover, studies have also correlated the action of histone methyltransferases (HMTs), particularly Mixed-Lineage Leukemia 1 (MLL1) protein, with the development and progression of ALL. MLL1 protein is known to methylate lysine 4 of H3 histone (H3K4) [91, 98, 99].

The first study carried out in this regard demonstrated that inhibition of G9a (with BIX01294) reduced the ability of infant ALL cells to migrate through an endothelial monolayer, an essential function for leukemia to progress and migrate to other tissues. Additionally, cells treated with this inhibitor showed nuclear enlargement with a more adherent phenotype than control cells. These findings suggest a solid connection between G9a function and the integrin VLA-4 (alpha4beta1), responsible for cell migration [100].

In addition to migration, other researchers investigated the effect of G9a blocking on the cell cycle, proliferation, and apoptosis of acute lymphoblastic leukemia T cells (MOLT-4 and Jurkat cell lines). G9a depletion directly regulated histone 3 lysine 9 (H3K9) methylation. This modulation produced effects such as inhibition of proliferation, induction of apoptosis, and even cell cycle arrest in the G0/G1 phase of the analyzed cells [101]. Our group also investigated the effect of G9a/GLP inhibition on ALL. *In silico* analysis revealed that both enzymes are upregulated in ALL samples when compared to control bone marrow samples. Interestingly, G9a/GLP inhibition by UNC0646 induced apoptosis in Jurkat cells, which was accompanied by increased expression of P53, TP73, BAX, and MDM4 [81].

A recent study investigated G9a/EHMT2 as a potential therapeutic target for T-ALL. Using CRISPR-Cas9 targeting the catalytic SET-domain and chemical inhibitors (BIX01294), they validated distinct approaches to target G9a in vitro, in vivo, and in 3D models. They focused on elucidating the role of G9a inhibitors as inducers of autophagic cell death. The results strongly suggested that in T-ALL, G9a suppression inhibits Glycogen Synthase Kinase 3 (GSK-3), demonstrating an effective epigenetic control on glycolytic pathways to deplete the metabolic requirement of T-ALL cells [102].

Although few studies are correlating the action of either G9a/GLP or GLP blocking separately with ALL, the data we have so far indicates that control of the G9a/GLP pathway represents a potential treatment for ALL [103]. In addition to being involved in the migratory potential, proliferative capacity, and control of cell death in ALL, G9a/GLP also appears to have a role in the development of CLL, as will be discussed below.

## Chronic lymphoblastic leukemia

CLL is a neoplastic disease characterized by a monoclonal expansion of mature small B lymphocytes (CD5^+^CD23^+^) in the peripheral blood, being the most common adult leukemia in the Western world [104–106]. It has an insidious development and is usually diagnosed in routine blood tests in asymptomatic individuals. CLL is more common in elderly patients, with increasing incidence while advancing age, representing more than 20 cases for every 100,000 inhabitants over 70 years of age. It is a disease with a very heterogeneous manifestation [107]. CLL patients need to start therapy only if with active or symptomatic disease, some cases remain with an indolent disease for many years and there is no need to treat [104, 108].

There is still no consensus on the leukemic cell origin. Some chromosomal aberrations have been demonstrated to often initiate the CLL [del(11q), del(13q), and trisomy 12], followed by additional mutations that can dictate the pathophysiologic role of the disease [109, 110]. Genetic profile studies indicate that it can originate from naïve B cells or even from splenic marginal zone B cells. In any case, there are two distinct profiles, classified by the presence or absence of mutation in Ig heavy-chain variable-region genes (IGHV), and considering all the aspects, possibly in addition to genetic rearrangements, epigenetic events are also necessary for B cells to become neoplastic [104].

Epigenetic mechanisms such as DNA methylations, histone modifications, and chromatin availability have already been described in CLL. Aberrant EZH2, which results in histone modifications, seems to affect the pathogenesis of the disease, though in a minimum fraction of CLL patients [111, 112]. One recent research was carried out to measure and compare epimutations rate on healthy B cells and CLL patient samples. The group primarily studied DNA methylation patterns. Their results presented an elevated epimutation rate in CLL cells with low cell-to-cell variability in contrast to healthy B cells [113].

Regarding epigenetic modulation associated with G9a/GLP, there are few studies related to CLL. Our group identified overexpression of GLP in samples from CLL patients, and its aberrant expression was associated with a poor prognosis. Additionally, we observed that G9a/GLP pharmacological inhibition markedly induced cell death in CLL cells, demonstrating the role of these methyltransferases as potential targets for treatment and progression control of CLL [80].

## Multiple myeloma

MM is a hematologic disease characterized by neoplastic proliferation of plasma cells with increased production of M protein. It originates with a monoclonal gammopathy of unknown significance to plasma cell leukemia in the bone marrow and may affect secondary organs causing anemia, kidney injury, hypercalcemia, and bone lesions [114, 115]. Although its etiology remains unknown, some factors such as exposure to radiation, agricultural toxins, and several viruses were associated with its development [114, 116]. MM accounts for 10% of hematological neoplasms. The average age at diagnosis is around 65 years. There is an interesting difference between incidence and outcome with increased risk for black people and men over women [116]. In general, the disease is preceded by the progression of monoclonal gammopathy of undetermined significance (MGUS) and smoldering multiple myeloma (SMM) [117, 118]. Importantly, some chromosomal abnormalities involving the immunoglobulin heavy chain switch region have already been related to the development of the MM [116]. Interestingly, alterations in KRAS and NRAS, factors associated with DNA repair (deletion 17p, TP53, and ATM), and MYC constitute risk factors for progression from SMM to MM [103].

RAS mutations, TP53 alterations and MYC translocations are oncogenic drivers that appear to be associated with progression from MGUS to MM [119]. The progression from MGUS to MM is also related to epigenetic mechanisms such as DNA methylation, change in the methylation pattern, and histone acetylation. Drug resistance, disease progression, and malignant cell plasticity are directly influenced by the epigenetic machinery. In the early stages, the main mechanism found is global DNA hypomethylation [117, 120]. In contrast, histone demethylation with a transcriptional increase of oncogenes is generally found in the later stages of the disease, promoting increased leukemic cell viability and clonal proliferation. This global hypomethylation event was recognized by some authors as a component that contributes to the progression from MGUS to MM, which enforces that epigenetic events must play an important role in the malignancy transformation [117, 120, 121].

A recent study investigated the interplay between G9a/EHMT2 expression and MM. This study used the comparison between malignant cells and Peripheral Blood Mononuclear Cells (PBMCs) to verify if there are significant differences between them. The authors also assessed patients’ overall survival from a database. In addition, they analyzed the role of G9a in the development of the disease as well as its influence on tumorigenicity. G9a upregulation was observed in several MM cell lines, and its increased expression was correlated with worsened disease outcomes. They demonstrated that G9a contributes to myeloma cell survival and proliferation by interacting with NF-κB pathway [76]. Complementarily, other researchers have investigated the outcome of in vitro inhibition of the G9a/GLP in MM cells. They demonstrated that this epigenetic pathway inhibition can induce cell apoptosis and G1-phase arrest. Those results provide evidence that G9a/GLP is responsible for tumor prolonged survival [120, 122, 123].

In vivo studies with specific chemical inhibition of G9a/GLP have already been designed using mice models. All results supported the premise that this therapeutic target achieved significant antitumor efficacy with a reduction in tumor burden and prolonged survival [120, 122]. Those authors highly suggest that G9a/GLP represents a promising therapeutic target strategy for MM patients [76, 117, 120].

## GLP and G9a as therapeutic targets

The growing knowledge of epigenetic modifications on cancer development and progression led to the development of epigenetic modifying agents targeting specific histones modifiers [124]. EZH2, DOT1L and G9a are some HMTs that are being explored as epigenetic targets. For example, some epigenetic modifying agents, including Tazemetostat, GSK126, and UNC199 were developed to control the oncogenic role of EZH2 [125]. Pinometostat represents a DOT1L inhibitor that has shown therapeutic potential for cases of acute leukemias involving mixed lineage leukemia (MLL) gene rearrangements [126]. The therapeutic strategy of epigenetic modulation could represent a considerable advance for personalized medicine. The fact that epigenetic modifications are reversible provides possibilities for the development of therapeutic approaches in which by targeting specific enzymes involved in histone modifications or DNA methylation, it is possible to alter the epigenetic landscape and restore normal patterns of gene expression [99, 125].

**Fig. 4:**
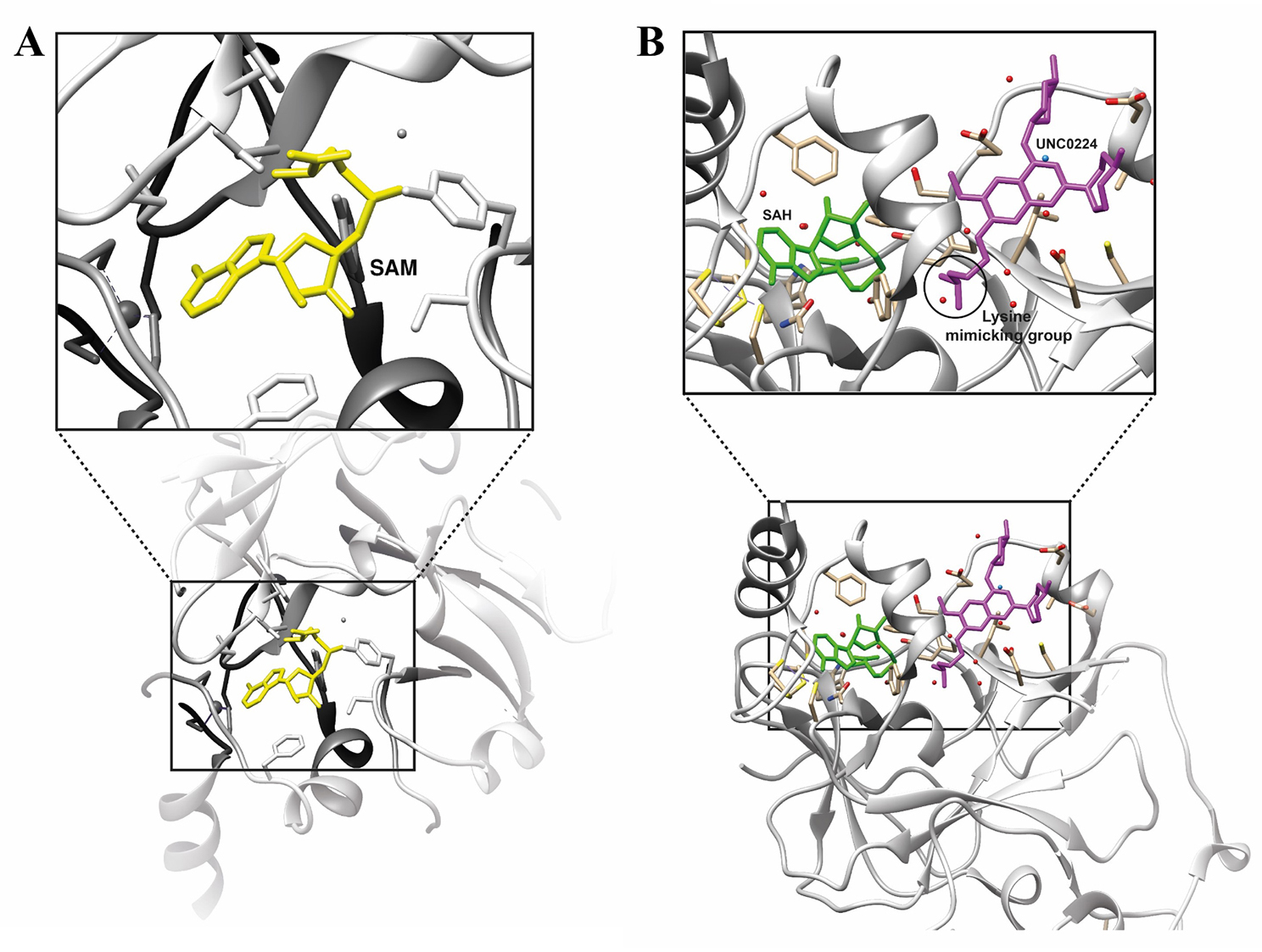
Crystal structure of G9a with cofactor SAM in yellow (PDB ID: 5JJ0) (**A**) and Crystal structure of G9a with a lysine-binding tunnel occupied by the substrate-competitive inhibitor UNC0224 (PDB ID: 3K5K) (**B**). SAH: S-adenosyl homocysteine, SAM: S-adenosyl methionine

In the last years, several GLP/G9a inhibitors have been developed, including BIX-01294, A-366, UNC0642, UNC0638, and DS79932728 among others [59, 77, 127–133]. They are classified into three main groups based on their binding modes: substrate competitive inhibitors, SAM (S-adenosyl -methionine) competitive inhibitors, and inhibitors with unidentified mechanisms (Table 1). While SAM competitive inhibitors prevent histone methylation by binding to SAM binding pocket, substrate competitive inhibitors occupy the substrate site of these enzymes, which promotes a better selectivity against the target [134] (Fig. 4).

**Table 1:**
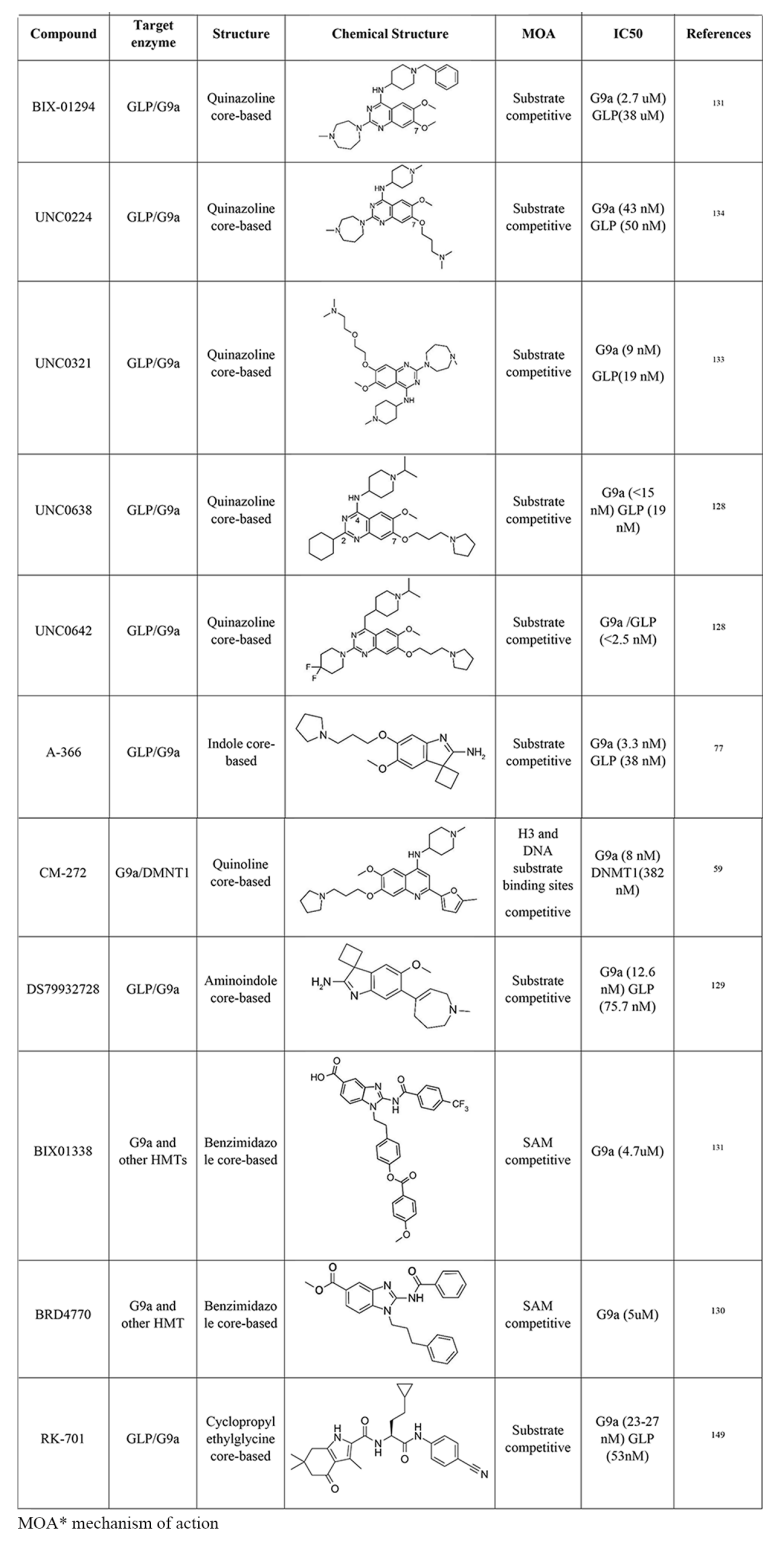
Epigenetic drugs targeting GLP/G9a

BIX-01294 (diazepin-quinazolin-amine derivative) was the first pharmacological GLP/G9a selective inhibitor described [133]. It is highly selective for G9a, but at higher concentrations, it also inhibits GLP. BIX-01294 showed anticancer activity by reducing cell proliferation and inducing apoptosis in glioma [135], bladder cancer [136], neuroblastoma [137], nasopharyngeal carcinoma cells [138], and diffuse large B-cell lymphoma cells [139]. It also induced apoptosis, cell cycle arrest, and upregulated the tumor suppressor protein p15 in acute T lymphoblastic leukemia cells [101]. A reduction in cell viability was reported in both human and murine MM cells in vitro. The same study showed that GLP/G9a inhibition in a murine model reduced tumor progression and sensitized MM cells to the proteasome inhibitors (PIs) action [120]. Similarly, inhibition of GLP and G9a using BIX-01294 increased type I interferon and Imatinib responsiveness in several Chronic Myeloid Leukemia (CML) cell lines [140]. Despite the promising results as an anticancer agent, BIX-01294 use displayed a poor separation of functional potency, and cell toxicity, which limits its use as a chemical probe [133, 141]. Further modifications to the structure of BIX-01294 have led to the development of new inhibitors, such as UNC0224 [127], UNC0321, UNC0638, and UNC0642 [129]. The development of these inhibitors, mainly UNC0638 and UNC0642, achieved high potency in vitro assays and excellent selectivity, compared to the results obtained with BIX-01294 [141].

When compared to BIX-01294, UNC0224 and UNC0321 presented a strong enzymatic inhibition profile but low cellular activity, probably related to permeability problems [127, 128]. On the other hand, UNC0638 [59] exhibited great GLP/G9a inhibition activity and induced apoptosis, suppressed migration, and tumor invasion in several cancers such as breast cancer [142], renal cancer [143], and ovarian cancer cells [144]. Unfortunately, the poor pharmacokinetic (pk) limited its application in animal studies.

Among the non-competitive inhibitors, UNC0642 [129] presented a higher selectivity for G9a/GLP, low cytotoxicity, and improved pk, being a promising candidate for animal studies. In vitro studies showed that UNC0642 treatment decreased cell viability and colony formation ability of CML cells [79]. Similar results were also observed in MM [76]. Additionally, G9a/GLP inhibition by UNC0642 during the expansion of TCR-engineered T cells increased its cytotoxicity against hepatocellular carcinoma [145].

The efforts to identify chemically distinct G9a inhibitors led to the discovery of the indole core-based molecule A-366, an H3 peptide-competitive inhibitor. In addition to a high inhibition potency and selectivity for G9a and GLP, A-366 showed significantly less cytotoxic effects when compared to other G9a/GLP inhibitors [146]. A-366 also displayed pro-differentiation effects on leukemia. After A-366 treatment, upregulation of cell differentiation markers in several leukemia cell lines was observed, accompanied by growth inhibition and decreased viability, especially in MV4;11, a cell line from acute myeloid leukemia. It also induced differentiation and decreased proliferation in acute promyelocytic leukemia (PML) cell line HL-60 [77].

CM-272 is the first-in-class dual inhibitor designed to inhibit both G9a and DNMT1 methyltransferase activity. It was shown that DNMT1 and G9a interact during the cell cycle to coordinate DNA and H3K9 methylation [147, 148], and their inhibition leads to the reactivation of tumor suppressor genes and inhibits cancer cell proliferation [23]. Interestingly, this new compound inhibited cell proliferation, promoted apoptosis, and induced the expression of interferon-stimulated genes on AML, ALL, and diffuse large B-cell lymphoma-DLBCL cell lines in vitro [130]. It also prolonged the survival of AML, ALL, and DLBCL xenogeneic models.

Recently, a new potent G9a/GLP inhibitor was identified. DS9932728 is an aminoindole derivative with good metabolic stability, and a high potency in inhibiting G9a (IC50 of 4.50 nM) and GLP (IC50 33.9 nM), showing promising results in inducing γ-globin production in monkeys [131]. This finding is particularly relevant for the search for new therapeutic options for the treatment of hemoglobinopathies, such as Sickle Cell Anemia and β-thalassemia. Importantly, reactivation of fetal globin expression was also observed in human erythroid cells and in mice after treatment with RK-701, a high-selectivity, low-toxicity small molecule inhibitor of G9a and GLP. The kinetic analysis suggests that RK-701 could be classified as a histone H3 substrate competitive inhibitor [149].

Evidence of G9a inhibition by niclosamide, a parasitic drug, was also reported [150]. Although first designed for treating tapeworm infection, niclosamide also displayed cytotoxic properties in malignant tumors, including lung cancer cells [151], adrenocortical carcinoma [152], and CML [153]. Interestingly, niclosamide was able to reduce G9a protein levels in a dose-dependent manner in DLBCL cell lines. These results were followed by modulation of autophagy-related genes expression and suppression of cell growth [150]. Up to date, there are no clinical studies using G9a/GLP inhibitors.

## Conclusions and future directions

Histone methyltransferase inhibitors are gaining significant attention as an innovative therapeutic option. However, despite their promising efficacy against multiple cancers in preclinical models, this potential cannot always be fully translated to the clinical context. This could be due to low/poor oral bioavailability - requiring continuous IV infusion, the requirement of high doses to exert the desired therapeutic effect, or high toxicity [154–156]. Thus, future research should focus on overcoming these challenges to enhance the therapeutic efficacy of epigenetic drugs. A strategy that could be used to overcome some of these challenges is combinatorial therapy. Combining epigenetic drugs with standard and/or targeted strategies could be a better option than monotherapy due to lower doses of each inhibitory agent to achieve the desired therapeutic effect, thus, limiting toxicities [155, 156]. Research should also focus on studying the combinations’ pharmacodynamics to explain why certain combination strategies may be effective while others may not. It is also necessary to focus on identifying particular patient subgroups that would benefit from a special combination regimen, as some clinical trials can be conducted with newly diagnosed patients, relapsed/refractory patients, or patients harboring some specific genetic alterations.

Many ongoing clinical trials are exploring the potential of the combinations of epigenetic drugs and other therapeutic agents in hematological malignancies. Pinometostat (EPZ-5676), a DOT1L inhibitor, was tested in children with relapsed or refractory acute leukemias harboring *MLL* (*KMT2A*) gene rearrangement (*MLL*-r) as monotherapy in phase I clinical trial (NCT02141828), but no objective responses were observed in this cohort [157]. Pinometostat was also tested in adult patients with *MLL*-r acute leukemias as monotherapy in phase I clinical trial (NCT01684150). Although the IV administration was generally safe in patients, the maximum tolerated dose was not established, and the results showed limited clinical efficacy as a single agent [154]. However, these results provided a basis for future combinatorial approaches. Hence, it will be tested, in combination with daunorubicin and cytarabine, in phase I/II clinical trial with patients with newly diagnosed *MLL*-r acute myeloid leukemia (NCT03724084). It will also be tested in patients with relapsed or refractory multiple myeloma in combination with dexamethasone and CC-92,480 (BMS-986,348) – an agent that promotes the degradation of the transcription factors Ikaros and Aiolos, which leads to apoptosis and immunomodulatory effects [158, 159] (NCT05372354). SNDX-5613, a potent and specific Menin-MLL inhibitor, will be tested in phase I clinical trial in patients with relapsed or refractory *MLL*-r acute leukemias harboring or mutations in *Nucleophosmin 1* (*NPM1*) gene (NCT04065399). Another Menin-MLL inhibitor that also will be tested in phase I clinical trials in patients with acute leukemias is JNJ-75,276,617, as a monotherapy (NCT04811560), and as a combinatorial agent with venetoclax and azacitidine (NCT05453903). Tazemetostat, an EZH2 inhibitor, is also being tested in clinical trials for a variety of solid tumors as a single agent or as a combinatorial agent, and it should not be long before clinical trials in hematological malignancies are carried out as well.

Despite the central role of G9a/GLP in controlling multiple oncogenic processes, specific inhibitors have not yet entered clinical studies at this point, mainly due to toxicity. Nevertheless, as knowledge regarding epigenetic drugs expands, it is expected that the accumulated clinical experience will lead to the development of strategies to accurately assess the therapeutic potential of targeting G9a/GLP in solid and hematological cancers.

## Data Availability

No datasets were generated or analysed during the current study.
